# Supplementary material for: Determinants of primaquine and carboxyprimaquine exposures in children and adults with Plasmodium vivax malaria
Source: Antimicrob Agents Chemother. Author manuscript; Available in PMC 2021 Oct 25. (PMC8522776; doi:10.1128/AAC.01302-21)
Supplement: Supplementary Materials [file EMS133267-supplement-Supplementary_Materials.pdf]

Supplementary Figure 1: time since previous dose versus the primaquine and carboxyprimaquine blood concentration. The dashed vertical lines show the 5th and 95th quantiles of the time since last dose.

Supplementary Figure 2: Primaquine and carboxyprimaquine log concentrations versus chloroquine and piperaquine log concentrations measured at the same timepoints. The dashed lines show a univariate linear regression to highlight the trend.

Supplementary Figure 3: Methemoglobin as function of the time since starting primaquine (black circles: 0.5 mg/kg target dose for 14 days; red circles: 1 mg/kg target dose for 7 days). We added random jitter to the time in days for visual clarity.

Supplementary Table 1: Estimated regression coefficients (standard errors) for the linear terms in the main models. NI: not included. Age is included in these models as a non-linear term and so it is excluded from this table (effects are shown in Figures 1 & 3). For the time to recurrence model, the coefficients and standard errors are given on the linear scale (hazard ratio is given by the exponential of the coefficient).

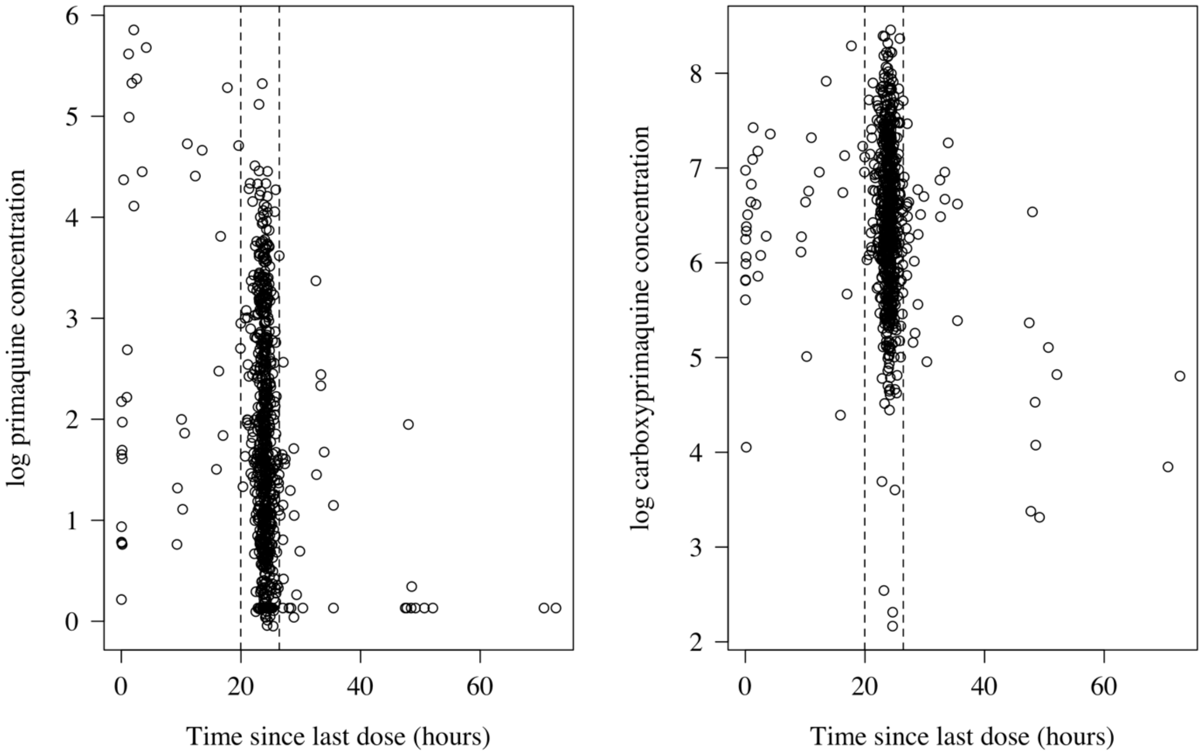

Supplementary Figure 1

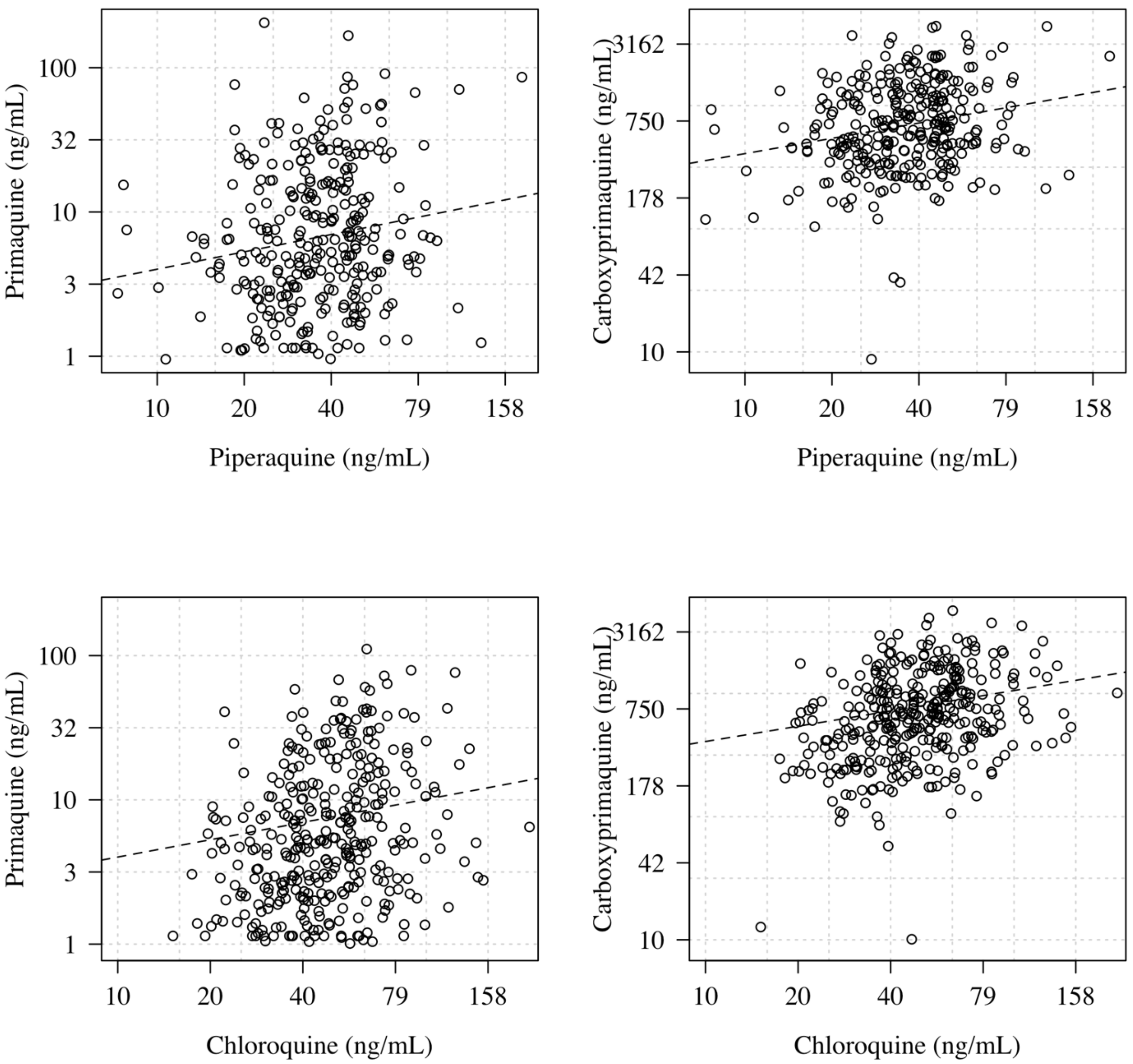

Supplementary Figure 2

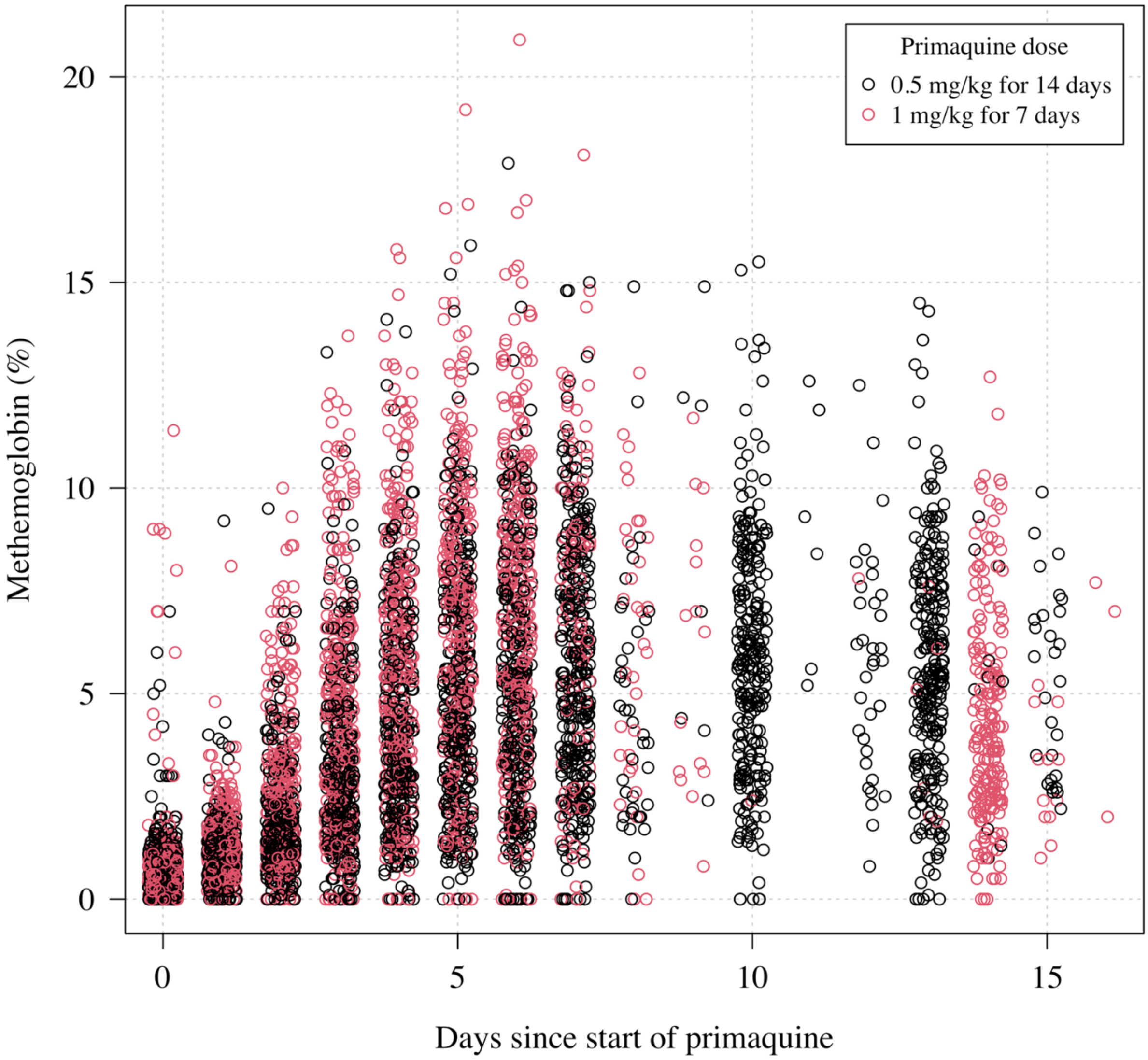

Supplementary Figure 3

Outcome variable	Regression type	Dose (mg/kg)	Partner drug	Days of primaquine	Fever clearance (days)	CYP 2D6 activity score < 1	G6PD deficiency	Methb (%)
PQ (log ng/mL)	linear	1.14 (0.06)	0.014 (0.03)	0.038 (0.047)	-0.017 (0.021)	NI	NI	NI
CPQ (log ng/mL)	linear	0.85 (0.040)	-0.020 (0.021)	0.042 (0.032)	-0.027 (0.014)	NI	NI	NI
log CPQ/PQ	linear	-0.28 (0.043)	-0.039 (0.022)	-0.0040 (0.035)	-0.0086 (0.016)	NI	NI	NI
Methb (%)	linear	3.14 (0.92)	NI	-1.11 (0.77)	NI	-1.21 (0.59)	-0.43 (0.86)	NI
Hb reduction (%)	linear	-2.43 (1.43)	-2.90 (0.73)	3.07 (1.13)	-2.10 (0.50)	NI	-12.0 (1.67)	NI
Time to recurrence	Cox proportional hazards	NI	-0.23 (0.24)	NI	NI	NI	NI	-0.09 (0.039)

Supplementary Table 1
